# A Nonoxidative Electrochemical Sensor Based on a Self-Doped Polyaniline/Carbon Nanotube Composite for Sensitive and Selective Detection of the Neurotransmitter Dopamine: A Review

**DOI:** 10.3390/s8128423

**Published:** 2008-12-18

**Authors:** Shah R. Ali, Rishi R. Parajuli, Yetunde Balogun, Yufeng Ma, Huixin He

**Affiliations:** Chemistry Department, Rutgers University. 73 Warren St. Newark, NJ 07102, USA; E-mails: shahali@andromeda.rutgers.edu; rparajul@pegasus.rutgers.edu; yufengma@email.pegasus.rutgers.edu;

**Keywords:** Boronic Acid, Dopamine Detection, Nonoxidative Sensor, Polyaniline, Carbon Nanotube, Parkinson's disease

## Abstract

Most of the current techniques for *in vivo* detection of dopamine exploit the ease of oxidation of this compound. The major problem during the detection is the presence of a high concentration of ascorbic acid that is oxidized at nearly the same potential as dopamine on bare electrodes. Furthermore, the oxidation product of dopamine reacts with ascorbic acid present in samples and regenerates dopamine again, which severely limits the accuracy of the detection. Meanwhile, the product could also form a melanin-like insulating film on the electrode surface, which decreases the sensitivity of the electrode. Various surface modifications on the electrode, new materials for making the electrodes, and new electrochemical techniques have been exploited to solve these problems. Recently we developed a new electrochemical detection method that did not rely on direct oxidation of dopamine on electrodes, which may naturally solve these problems. This approach takes advantage of the high performance of our newly developed poly(anilineboronic acid)/carbon nanotube composite and the excellent permselectivity of the ion-exchange polymer Nafion. The high affinity binding of dopamine to the boronic acid groups of the polymer affects the electrochemical properties of the polyaniline backbone, which act as the basis for the transduction mechanism of this non-oxidative dopamine sensor. The unique reduction capability and high conductivity of single-stranded DNA functionalized single-walled carbon nanotubes greatly improved the electrochemical activity of the polymer in a physiologically-relevant buffer, and the large surface area of the carbon nanotubes increased the density of the boronic acid receptors. The high sensitivity and selectivity of the sensor show excellent promise toward molecular diagnosis of Parkinson's disease. In this review, we will focus on the discussion of this novel detection approach, the new interferences in this detection approach, and how to eliminate these interferences toward *in vivo* and *in vitro* detection of the neurotransmitter dopamine.

## 1. Introduction

Tremendous efforts have been made over the last thirty years to detect biogenic amines, especially dopamine, for *in vivo* studies [1-6]. Information regarding the temporal fluctuations of the dopamine concentration in the brain is critical for understanding its widespread effects as a neurotransmitter. Moreover, abnormal dopamine transmission has been linked to several neurological disorders, e.g., schizophrenia, Huntington's disease and Parkinson's disease. As Parkinson's Disease (PD) is characterized by a severe depletion of the *in vivo* dopamine pool [3], the ability to sensitively and selectively measure the concentration of the neurotransmitter dopamine could potentially be used for molecular diagnosis of Parkinson's disease. A clinical, analytical screening method to diagnose PD does not exist. The ability to physiologically monitor the concentration change of dopamine could also benefit the design of therapeutics and evaluation of their therapeutic efficacy towards PD [7].

Dopamine (DA) can be easily oxidized electrochemically at conventional electrodes, which have been used to detect the neurotransmitter in both *in vitro* and *in vivo* environments [1-6, 8, 9]. An electrochemical approach has many advantages over other methods: the electrodes can be made extremely small and can be conveniently implanted in living organisms with minimal tissue damage and, because the response is fast, the neurotransmitter can be monitored in real time. Adams pioneered the use of electroanalytical sensors to detect chemical changes in brains in the 1970s [10]. Since then substantial research has been performed along this direction. However, there remain a number of challenges with the electrochemical method due to the nature of the oxidative reaction of dopamine. One of the primary challenges is that the concentration of dopamine in the extracelluar fluid of the caudate nucleus is extremely low (0.01–1 µM for a healthy individual and in the nanomolar range for patients with Parkinson's disease), while the concentrations of the main detection interferents, e.g., ascorbic acid (AA), are usually several orders of magnitude higher (ascorbic acid levels are generally within 0.1–0.6 mM) [1, 11-14]. Furthermore, the interferents undergo oxidation within the same potential window as dopamine. As a result, an overlapping voltammetric response for the oxidation of a mixture of DA and AA is obtained. In addition, a large overpotential and electrode fouling by oxidation products cause additional difficulties in the detection of DA. Inspired by the fact that enzymes can react selectively with their cognate substrates, efforts have been made to immobilize dopamine-specific enzymes, such as polyphenol oxidase, onto the electrodes to increase the selectivity [15]. However, the main interferent, ascorbic acid, still hinders severely the accurate detection of dopamine because the oxidized dopamine product (dopamine-*o*-quinone), produced from either direct oxidation at the electrode or by the enzymes immobilized on the electrode, can catalytically oxidize ascorbic acid nearby to regenerate dopamine that becomes available again for oxidation [16].

A variety of surface modification approaches has been exploited to resolve these problems. One type of surface modification exploited the opposite polarities of DA and AA [17-24]. The modified film on the electrode can attract one and repel the other. Another set of surface modification is to incorporate electrochemical mediators, including some novel nanomaterials, such as Au nanoparticles and carbon nanotubes, in the modified layer [25-28]. These approaches have shown great success in selectivity by inhibiting the interference reactions or promoting dopamine oxidation at different potentials. Special efforts have been paid to promote AA oxidation ahead of DA, which can effectively avoid the dopamine regeneration problem. Simultaneous detection of AA and DA, and even uric acid, were demonstrated [19-24]. Furthermore, due to the largely reduced overpotential and the existence of the surface modification layer, the electrodes show excellent stability against electrode fouling [20, 27]. However, the detection limits in most of the reports are larger than 10 nM, which is not sensitive enough for diagnosis of Parkinson's disease.

New materials, such as carbon nanotubes [29] and boron-doped diamond [30] have been used to fabricate novel electrodes for DA detection. New techniques have also been developed in an attempt to solve the aforementioned problems, such as hybrid amperometric and conductometric measurements based on conducting polymer nanojunctions [31], fast scan cyclic voltammetry (FSCV) and the relevant data treatments [1].

FSCV has been the most successful approach for *in vivo* detection of catecholamine fluctuations because of its high sensitivity and selectivity [1, 11, 12, 32, 33]. In this technique, the potential applied to a carbon-fiber microelectrode is cycled at scan rates faster than 100 V/s. Compounds with slower electron transfer rates, like ascorbic acid, can be distinguished very easily from biogenic amines at high scan rates because the oxidation of ascorbate is drawn out to high potentials. To a certain extent, this technique also avoids the problems of dopamine regeneration and electrode fouling due to the fast turn-around potential applied to the electrodes [11, 34]. More recently, the carbon-fiber microelectrodes have been modified by covalent attachment of molecules via diazonium salt reduction. For example, the electrodes were modified with anionic functional groups such as carboxyphenyl [35], phenylacetate [36], or sulfobenzene [37]. Electrodes modified in this way show further improved sensitivity and selectivity to catecholamines over ascorbic acid without slowing down the response time of the detection.

However, at high scan rates the majority of the current detected at the carbon-fiber working electrode is a nonfaradic current that arises from charging of the double layer. Even though techniques to subtract this large background current have been developed [38, 39], FSCV is typically only used to observe concentration changes over the time course of a minute. This is because the background current is only stable for a brief time. Therefore, it is not possible to measure basal concentrations of dopamine due to the differential nature of the technique. Readers interested in this area are referred to an extensive review about current development in detection of neurotransmitters and monitoring their rapid concentration dynamics within neural tissue [1].

While oxidative dopamine sensors are the most widely used platforms for the electrochemical detection of dopamine and are among the most successful in elucidating fundamental information concerning physiological and neurological transmission of dopamine, approaches that could alleviate all the afore-mentioned problems are attractive. One of the approaches is an electrochemical detection method that does not rely on the direct oxidation or reduction of dopamine itself (refers to *nonoxidative* dopamine sensors).

The first non-oxidative dopamine sensor was reported by Beni *et al.* [40]. They selectively detected dopamine electrochemically at the interface between two immiscible electrolyte solutions. The two liquid phases consisted of an aqueous layer in which both ascorbate and dopamine were dissolved, and an organic layer formed by 1,2-dichloroethane. The crown ether dibenzo-18-crown-6 was used as an ionophore that could transport the positively charged neurotransmitter dopamine (the amine group from dopamine binds the crown ether) from the aqueous layer to the organic layer. The transfer of ionized dopamine across the interface is accompanied with a potential difference, which produced a current that can be measured in a voltammogram. As ascorbate cannot be transported to the organic layer since it lacks affinity to the ionophore, it could not contribute to the measured amperometric signal and therefore the interference from ascorbate was eliminated. Strawbridge *et al.* detected the oxidation of phenylboronic acid, a diol-binding molecule, whose oxidation potential shifted upon binding with dopamine. AA interference was eliminated because AA did not interact with phenylboronic acid [41]. Recently this idea was applied by Wu *et al.* to increase the detection sensitivity by modification of the electrodes with a layer of multiwalled carbon nanotubes that were covalently linked to a phenylboronic acid derivative through carbodiimide coupling [42]. Fabre *et al.* extended this principle by using a polyaniline derivative, poly(anilineboronic acid), which preferentially binds dopamine over ascorbic acid by exploiting boronic acid-diol chemistry [43]. Formation of the resulting boronate ester changed the electrochemical behavior of the polyaniline backbone, which was used to transduce the binding event. However, the detection limits of these techniques were in the micromolar range, and therefore these detection methods are not sensitive enough for molecular diagnosis of Parkinson's disease.

Recently, we draw on the findings that single-stranded DNA (ss-DNA) can disperse bundled carbon nanotubes in aqueous solution, resulting in helical wrapping of single-walled carbon nanotubes by ss-DNA (ss-DNA/SWNTs) [44, 45]. The solubility and the unique surface properties of the ss-DNA/SWNTs permit “*in-situ*” electrochemical polymerization of 3-aminophenylboronic acid monomers to produce poly(anilineboronic acid)/ss-DNA/SWNT nanocomposites [46]. The nanocomposite shows excellent properties through synergistic effects of the component materials. The electrocatalytic reductive ability of the ss-DNA/SWNTs and their strong interaction with the polyaniline backbone significantly improved the stability of poly(anilineboronic acid) (PABA) [47]. The conductivity and electrochemical activity in neutral solution were greatly enhanced. The large surface area of the carbon nanotubes greatly increased the density of the boronic acid functional groups available for sensitive detection of the target analyte. Taking advantage of these remarkable properties of the nanocomposite, we developed a non-oxidative dopamine sensing approach, with which dopamine concentrations as low as 1 nM were detected with cyclic voltammetry and 40 pM with differential pulse voltammetry [48]. In this review, we will summarize our recent works along this line. First we will discuss the unique roles of carbon nanotubes in enhancing the performance of the conducting polymer, poly(anilineboronic acid), followed by a discussion of the dopamine transduction mechanism and ascorbic acid interference mechanism in this non-oxidative approach. Finally, we will discuss how to eliminate the interference of ascorbic acid toward the goal of molecular diagnosis of Parkinson disease.

## 2. Development of a nonoxidative dopamine sensor based on a nanocomposite of carbon nanotube/poly(anilineboronic acid)

### 2.1. Carbon nanotube/poly(anilineboronic acid) composites, multiple roles of single walled carbon nanotubes dispersed and functionalized by single stranded DNA

Conducting polymers are attractive for sensor applications because their electronic and electrochemical properties are highly sensitive to molecular interactions, which provide excellent signal transduction for molecular detection [49]. Among conducting polymers, polyaniline is unique since it is environmentally stable and easy to fabricate. It has been applied widely in chemical sensors [50-52] but not as much in biosensors [53-55]. The reason is that native polyaniline is neither electrochemically active nor conductive in neutral solutions, which is a standard prerequisite for biosensor applications. It is also limited both in the variety of molecules that can be detected and in the selectivity of the detection. Major breakthroughs in this field were the discoveries of self-doped polyaniline [56-59] and polyelectrolyte-anion-doped polyaniline [54, 55, 60], which brought polyaniline into the biosensor field due to the improved redox activity and conductivity in neutral pH solutions. However, compared to the parent polyaniline, the electrochemical activity, conductivity, and the chemical and mechanical stabilities of both self-doped polyaniline and bulky polyelectrolyte-doped polyaniline are greatly reduced, due to steric effects.

**Scheme 1.**
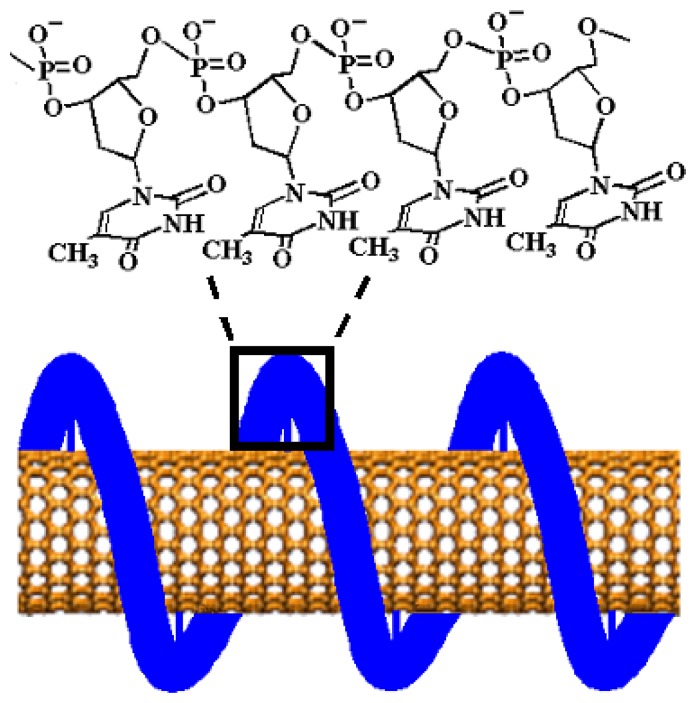
ss-DNA wrapped single walled carbon nanotube (SWNT). (The schematic is only a graphical presentation and does not represent the precise way ss-DNA binds on SWNTs.) (Reproduced with permission from the American Chemical Society [46].)

We found in this work that the stability of a self-doped polyaniline, poly(anilineboronic acid), is greatly improved when it is polymerized *in-situ* with ss-DNA-wrapped single walled carbon nanotubes (ss-DNA/SWNTs) (see Scheme 1). The redox properties of the polyaniline backbone are conserved in neutral solutions (pH 7.4), and the sensitivity for biomolecular detection is significantly enhanced. We found that the ss-DNA/SWNTs performed multiple roles in the greatly improved properties of the self-doped polyaniline both during and after the polymerization, which makes this work unique compared to previously reported conducting polymer/carbon nanotube composites [61-67]. First, they acted as effective *catalytic molecular templates* during polymerization of the self-doped polyaniline to increase the polymerization speed and improve the quality of the polymer. Second, they functioned as novel *active stabilizers* after the polymerization. One difficulty in working with self-doped polyaniline is that the fully oxidized pernigraniline form is not stable in aqueous solution and is difficult to completely reduce to the partially oxidized emeraldine and fully reduced leucoemeraldine states [55, 68-71]. In our case, pernigraniline was readily reduced in the electrochemical experiments due to the electrocatalytic reductive ability of ss-DNA/SWNTs [72, 73]. This ease in reduction significantly enhanced the stability of the film. Furthermore, the ss-DNA/SWNTs also acted as *conductive* polyanionic doping agents in the resulting polyaniline film that showed enhanced conductivity and redox activity. Finally, the large surface area of the carbon nanotubes greatly increased the density of the functional groups available for sensitive detection of the target analytes.

#### 2.1.1. Increased electrochemical polymerization speed: ss-DNA-SWNTs acted as catalytic molecular templates

**Figure 1.**
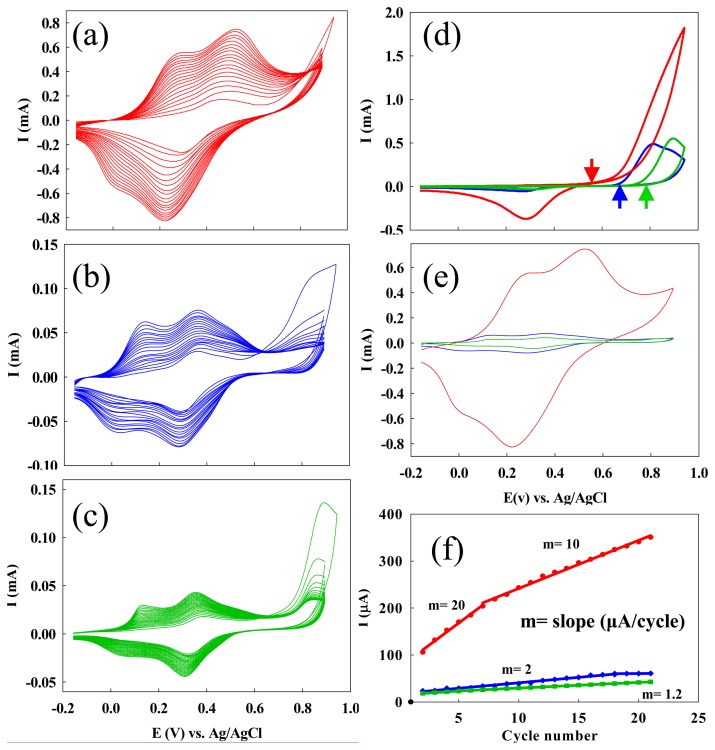
Cyclic voltammograms recorded during the electropolymerization of 3-aminophenylboronic acid on electrodes from the second cycle to 21st cycles at **(a)** ss-DNA/SWNT-modified **(b)** 2-aminoethanethiol- SAM-modified, and **(c)** double stranded-DNA-modified gold electrodes. Potential scan rate: 100 mV s-^1^. **(d)** The first cycles of the cyclic voltammograms in (a), (b) and (c). **(e)** The last (21^st^) cycles of the cyclic voltammorams in (a), (b) and (c). **(f)** The faradic current of the second oxidation peak of PABA on the modified electrodes as a function of cycles. (m is the slope of the fitted lines, in μA/cycle units.) (Reproduced with permission from the American Chemical Society [46].)

A gold (Au) electrode was first modified with a self-assembled monolayer (SAM) of 2-amino-ethanethiol so that the ss-DNA/SWNTs could adsorb onto the electrode. Poly(anilineboronic acid) (PABA) was then deposited onto the modified Au substrate by sweeping the electrochemical potential from -0.16 to 0.94 V (versus Ag/AgCl) in 0.05 M 3-aminophenylboronic acid monomer, 0.04 M KF, 0.5 M H_2_SO_4_. It was reported that the presence of F^-^ in the monomer solution could decrease the potential required for the polymerization of 3-aminophenylboronic acid [59, 74]. Thus, its addition minimized overoxidation of the polyaniline backbone. After the third cycle, the polymerization potential was decreased to 0.89 V to further reduce the possibility of overoxidizing the polyaniline backbone. Continued cycling of the potential resulted in repeated deposition of PABA onto the electrode surface with a slightly lower deposition rate after the first seven cycles (Figures 1a and 1f).

Figure 1a shows the CV curves during the polymerization. For comparison, the CV curves that were obtained without immobilization of ss-DNA/SWNTs on gold surfaces are shown in Figure 1b. To clearly read the initial potentials for the polymerization and to compare the polymerization current, we illustrated the first cycles for the electrodes with different modifications in Figure 1d. The large irreversible anodic peak observed in the first cycle belongs to the polymerization of 3-aminophenylboronic acid. We found that the monomer polymerized more readily on the ss-DNA/SWNTs-modified electrode, indicated by a negative shift (about 150 mV) in the initial polymerization potential (indicated by the arrows in Figure 1d). The maximum current for the polymerization was six times higher in the presence of ss-DNA/ SWNTs, providing further support for this point (Figure 1d).

In subsequent cycles, two pairs of peaks appeared (Figures 1a and 1b), which corresponded to the redox reactions of the polyaniline backbone: its transformation from insulating protonated leucoemeraldine to conducting protonated emeraldine, and then from conducting protonated emeraldine to insulating pernigraniline [70]. Figure 1e displays the CV curves for the last (21^st^) cycles, which clearly shows that the amount of PABA deposited on the ss-DNA/SWNTs modified electrodes was significantly larger than the electrode without ss-DNA/SWNTs modification. Figure 1f shows the current (the faradic current for the transformation from emeraldine to pernigraniline) as a function of the CV cycle numbers for each of the electrodes with different modifications. Without ss-DNA/SWNTs, the faradic current grew in intensity during the first 17 cycles and leveled off afterwards (Figure 1f), indicating that PABA was deposited onto the electrodes only in the initial stages of the experiment. At the ss-DNA/ SWNT modified electrodes, the PABA current increased during all cycles, with a slower increase rate after the 7^th^ cycle. From the slopes of the current vs. cycle number in Figure 1f, we can estimate the current increase speed, which is ten times faster than that at the electrode without ss-DNA/SWNTs. After seven cycles, the current increase rate lowered to 10 μA per cycle, which is still five times higher than that at the electrode without ss-DNA/SWNTs, where the rate remained 2 μA per cycle throughout. We attribute the faster deposition in the first seven cycles, especially in the first cycle, to the pre-concentration of the monomers by the carbon nanotubes and to the larger surface area conferred to the electrode by the ss-DNA/SWNTs.

We also used UV-Vis-Near IR spectroscopy to monitor *in-situ* the chemical polymerization process of 3-aminophenylboronic acid monomers (ABA) in the presence and absence of ss-DNA/SWNTs. We found that the polymerization process can be up to 4,500 times faster when ABA was polymerized in the presence of ss-DNA/SWNTs [75]. We attributed this remarkable catalytic behavior of the ss-DNA/SWNTs to the electronic interaction between the monomers and ss-DNA/SWNTs. ABA monomers pre-concentrated along the carbon nanotubes and formed SWNTABA complexes, which act as the polymerization precursors. Due to the electron richness of the ss-DNA/SWNTs, the ABA monomers had higher electron density, which greatly facilitated the polymerization. During the electrochemical polymerization, we found a negative shift (about 150 mV) in the initial polymerization potential (indicated by the arrows in Figure 1d), suggesting that the catalytic effect of ss-DNA/SWNTs also occurs during the electrochemical polymerization process [46].

#### 2.1.2. Improved conductivity: ss-DNA-SWNTs acted as a unique conductive doping agent

**Figure 2.**
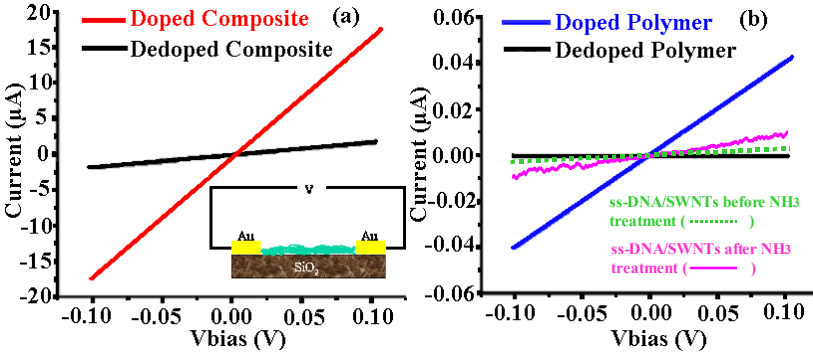
Typical I-V characteristic curves of the (a) PABA-ss-DNA/SWNTs composite film, (b) PABA alone, and ss-DNA/SWNT alone before and after treatment with NH_3_. (Reproduced with permission from the American Chemical Society [47].)

A prerequisite for continuous electrochemical deposition of polymer film is that the produced film must be conductive in each cycle of deposition; otherwise, the deposition would self-terminate. The redox current of the polyaniline backbone increased much faster on the electrode with ss-DNA/SWNTs than at the electrodes with ds-DNA and with the 2-aminoethanethiol monolayer only, respectively (Figures 1a, 1b, 1c and 1f). Figure 1e clearly shows that more PABA was deposited on the electrode with ss-DNA/ SWNTs. We attribute the increase in deposition in the subsequent cycles to the increased surface area of the electrodes imparted by the carbon nanotubes and to the better conductivity of the resulting nanocomposite film. Although we could not characterize the conductivity and the molecular structure of PABA in the composite on the gold electrode formed by electrochemical polymerization, we have characterized the composites fabricated by *in-situ* chemical polymerization of ABA in the presence or absence of ss-DNA-SWNTs [47, 76]. Figure 2 shows that the conductance of the composite in the doped state is 385 ± 6 higher than the sum of the conductance of pure PABA and ss-DNA/SWNTs alone [47]. We think the ss-DNA/SWNTs acted as a conductive doping agent, which caused the film to be more conductive and electrochemically active compared to the PABA film doped with a nonconductive polyanionic doping agent (i.e. ds-DNA) and small doping agents [77].

**Figure 3.**
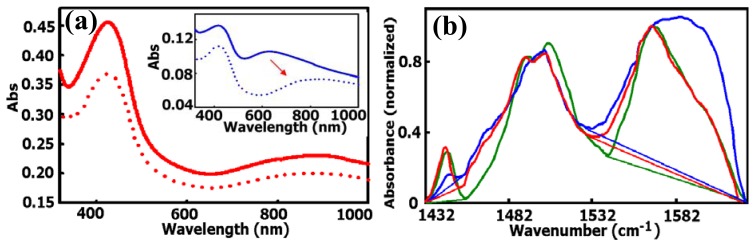
(a) UV-vis spectra of the PABA/ss-DNA/SWNT composite (red) and the pure PABA (inset, blue) before (-) and after (⋯) treatment with NaBH_4_; (b) FTIR spectra of the composite (red) and the pure PABA (blue) and the pure PABA after reduction with NaBH_4_ (green). The spectra were normalized with the 1570 cm^-1^ peak. Baselines for the absorption height ratio measurements are shown. (Reproduced with permission from the American Chemical Society [47].)

The better conductivity of the nanocomposite film is also due to the improved conductivity of the PABA component [47, 76]. Recently, we demonstrated that the contact resistance between carbon nanotubes can be largely decreased by *in situ* polymerization of a thin skin of PABA around and along the ss-DNA/SWNTs. The conductivity of the composite film after percolation can be two magnitudes higher than that of network films prepared from ss-DNA/SWNTs alone [76]. We have used UV-Vis-Near IR and FTIR spectroscopy (Figure 3) to characterize the PABA produced by chemical polymerization of 3-aminophenylboronic acid in the absence and presence of ss-DNA/SWNTs. While neat PABA existed in the nonconductive and degradable pernigraniline state of polyaniline, with a shorter conjugated length, PABA obtained with ss-DNA/SWNTs absorbs light at a longer wavelength and exists in the more conductive and stable emeraldine state of polyaniline [47, 76]. In fact, similar results were reported in the fabrication of conducting polymer nanowires or nanotubes using polycarbonate nanopores as templates [78-80]. According to these reports, conducting polymers preferentially nucleated and grew on the walls of the nanopores due to the coexistence of anionic sites and hydrophobic components on the pore walls. The resulting conducting polymer nanotubes normally had higher conductivity than conducting polymers produced by traditional methods due to the longer conjugated structures and better orientation of the polymer chains [78-80]. Therefore we conclude that the role of the ss-DNA/SWNTs not only includes increased polymer growth rate, as described above, but also facilitation of head-to-tail coupling during polymer growth [81], resulting in PABA with longer conjugated molecular structures. The ss-DNA/SWNTs acted as unique conductive doping agents, so that a highly conductive composite film was modified on the electrode surface, which greatly helps in developing a sensor with high sensitivity.

#### 2.1.3. Enhanced Stability: ss-DNA/SWNTs acted as active stabilizer

**Figure 4.**
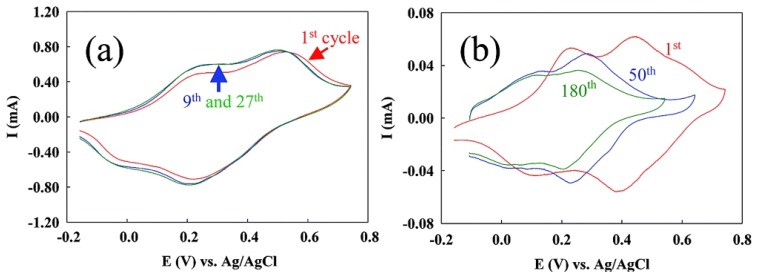
Successive cyclic voltammograms of poly(anilineboronic acid) in H_2_SO_4_ solutions on electrodes (a) with ss-DNA/SWNTs and (b) without ss-DNA/SWNTs; potential scan rate: 100 mV s^-1^ (Reproduced with permission from the American Chemical Society [46].)

Toward the goal of biosensor applications, which require operation under physiological conditions, the deep green PABA film obtained at the ss-DNA/SWNTs electrode was first stabilized in 0.5 M H_2_SO_4_ and then in 0.01 M phosphate buffered saline (PBS, pH 7.4) by sweeping the potential between 0.04 and 0.79 V until the cyclic voltammetry (CV) curves were stabilized. Interestingly we found that the stability of the resulting films was also increased by the presence of ss-DNA/SWNTs, as is indicated by the fact that the CV curves were easily stabilized in both H_2_SO_4_ (Figure 4a) and PBS for the PABA on the ss-DNA/SWNTs modified electrodes. In the absence of the ss-DNA/SWNTs, the current decreased continuously upon cycling, and the oxidation and reduction peaks of the polyaniline backbone also shifted significantly to negative potentials (Figure 4b). Figure 4b shows that after 180 cycles, the redox peaks of polyaniline are still decreasing and the peak positions are still shifting. Holding the film at -0.3 V for 5 minutes prior to the scan caused the CV response to be recovered to a certain extent. This behavior was often observed when the parent polyaniline film was cycled in higher pH solutions [55, 68], and it was attributed to the inability to reduce the fully oxidized pernigraniline completely over the timescale of the cyclic voltammetry experiment. On the contrary, with the ss-DNA/SWNTs on the electrode, the CV curve of the 9^th^ cycle is almost identical to the curves obtained from previous cycles, indicating that the film was stabilized by the 9^th^ cycle. We ascribe this enhanced stability to the synergistic interaction of ss-DNA and SWNTs, which results in the electrocatalytic reductive ability of the ss-DNA/SWNTs [72, 73]. Different from carbon nanotubes alone, ss-DNA-wrapped carbon nanotubes are surprisingly effective electron donors, instead of electron acceceptors [61, 82], and can be readily oxidized by strong oxidants such as KMnO_4_ and K_2_IrCl_6_ [72]. Recently the electrocatalytic reduction ability of the ss-DNA/SWNTs was demonstrated in the study of electrochemical oxidation of Ru(bpy)_3_^2+^. The ss-DNA/SWNTs reduced electrogenerated Ru(III) during electrooxidation of chemical Ru(bpy)_3_^2+^ [73]. Polyaniline has three states, and the fully oxidized pernigraniline state is not stable in aqueous solutions and is difficult to fully reduce. Both these characteristics could cause instability of the polyaniline film [55, 68-71]. Since the electropolymerization potential of 3-aminophenylboronic acid is more positive than the oxidation potential of the emeraldine to pernigraniline transition, the synthesized polymer naturally is in the fully oxidized pernigraniline state. However, due to the presence of ss-DNA/SWNTs, the pernigraniline produced during the potential cycling was readily reduced to the stable emeradine state, just like the electrogenerated Ru(III) was reduced during the electrooxidation of Ru(bpy)_3_^2+^. Therefore, ss-DNA/SWNTs greatly decreased the possibility of the polyaniline backbone's degradation. They also helped maintain the electrochemical activity of the film in the timescale of potential scanning. Without the ss-DNA/SWNTs, the only way to reduce the pernigraniline is to shift the potential to more negative values or to increase the time for reduction. Furthermore, the redox potentials of the polyaniline backbone kept shifting to more negative values in the absence of carbon nanotubes, which would make formation of pernigraniline easier and thus increases its quantity during the potential cycles. Therefore, even more serious degradation is induced in the absence of ss-DNA/SWNTs.

### 2.2. Non oxidative electrochemical detection of dopamine with enhanced sensitivity: ss-DNA-SWNTs increased effective electrode surface area

The great stability of the nanocomposite film and its electrochemical activity in pH 7.4 PBS allow us to use the self-doped polyaniline for sensitive detection of biomolecules in solutions approximating physiological conditions. After the composite film was stabilized in 0.01 M PBS (pH 7.4) solutions, various concentrations of dopamine were added into the electrochemical cell and the electrochemical current of the polyaniline backbone was measured. After each addition, the potential was swept between 0.04 and 0.79 V until the cyclic voltammograms (CV) were stable.

**Figure 5.**
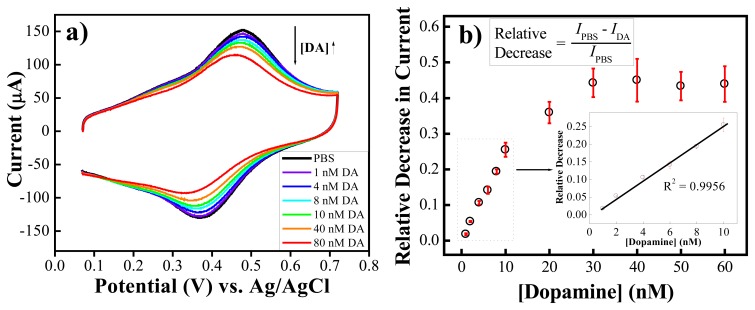
(a) Cyclic voltammograms of the composite in PBS and in the presence of different concentrations of dopamine; (b) a correlation curve of the data presented in 1a (n=5). Potential scan rate: 100 mV·s^-1^. (Reproduced with permission from the American Chemical Society [48].)

**Scheme 2.**
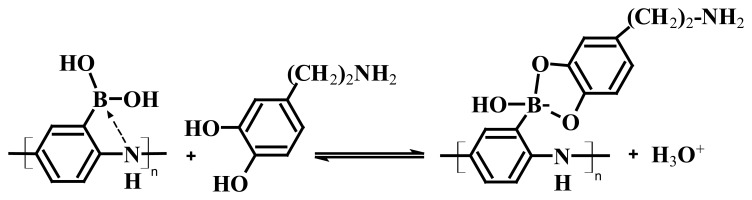
Complexation between dopamine and poly(anilineboronic acid) (Reproduced with permission from the American Chemical Society [48].)

Figure 5a shows the stable CV curves of the PABA/ss-DNA/SWNTs composite in PBS solution (pH 7.4) before and after adding different concentrations of dopamine. The CV curves show two redox couples centered at 0.25 V and 0.45 V (vs. Ag/AgCl), corresponding to the transition of the polyaniline backbone from the fully reduced leucoemeraldine state to the partially oxidized emeraldine salt state, and from the emeraldine salt state to the fully oxidized pernigraniline state, respectively. Addition of dopamine did not affect the redox peak potentials of the polyaniline backbone. However, the faradic current of the composite's anodic peak at 0.45 V decreases with increasing concentration of dopamine, which suggests that direct oxidation of dopamine on the electrode did not occur. Rather, the diol of dopamine chemically bound the boronic acid groups along the polyaniline backbone, which influenced the electrochemical acitivity of the polyaniline backbone (Scheme 2).

It is well known that native polyaniline is not conductive or electrochemically active in neutral pH solutions [43]. The boronic acid moieties extend the electrochemical activity and the conductivity of polyaniline towards higher pH due to the strong intra- or inter-chain tetrahedral boron–nitrogen interactions which stabilize the protonated emeraldine form at pH 7.4 [83, 84]. This self-doping process preserved to a large extent the electrochemical activity and conductivity of polyaniline in neutral pH solutions [59].

The high binding affinity between dopamine and boronic acid can affect the electrochemistry of the polyaniline backbone in different and seemingly divergent modes, and thus require clarification. On one hand, the conversion of the boronic acid to the boronate ester complex along the polyaniline backbone interrupts the intra- or inter-chain tetrahedral boron–nitrogen interactions, which *decreases* the self-doping and therefore the electrochemical activity of the polymer in neutral solutions. Furthermore, the steric effect of the formed anionic ester also hinders the electrochemical activity of the polyaniline backbone [85]. This is because oxidation and reduction of polyaniline during cyclic voltammetry are accompanied by conformational changes of the polymer backbone, which become less energetically favorable as large molecules are introduced along the backbone.

On the other hand, formation of the boronate complex eliminates the electron withdrawing nature of the boron's vacant *p*-orbital and instead, leads to an increase in the electron donating ability of the boron in the boronate substitutent groups. Increasing the electron donating ability of the substitutent is expected to stabilize the acid form of the quinone diimine group along the polymer backbone, a trademark of the conductive form of polyaniline (emeraldine salt), which means an enhanced self-doping ability. Therefore, the electrochemical activity of the polyaniline backbone should be *enhanced* upon binding. It becomes apparent that these two effects on the electrochemical activity of the polyaniline backbone offset one another. The net effect depends on the relative magnitude of the influences.

These multifaceted effects on electrochemical activity were observed recently in a boronic acid-substituted bipyridine Fe(II) complex [85]. Fabre *et al.* found that the apparent formal potential of the ferrocene/ferrocenium redox couple decreased and the redox current increased upon formation of the electron-donating boronate anion structure with F^-^, while with fructose an increase of the formal potential and decrease of redox current were observed. The authors ascribed these contrary changes to the different steric effects imparted by the F^-^ and fructose to the pyridine backbone in spite of their similar abilities to convert the boronic acid groups from electron withdrawing to electron donating substituents. Upon binding of dopamine, we found that the redox current decreased, suggesting that the steric effect of the formed anionic ester played the more important role. The resulting ester hindered the electrochemical activity of the polyaniline backbone, which is in agreement with the report by Fabre *et al.* [85].

It was reported that formation of the anionic ester could reduce the K_a_ of the protonated quinone diimine groups in the polyaniline backbone, and reduction of the K_a_ caused a positive shift in the potential of the electrochemical conversion of emeraldine to penigraniline. Shoji *et al.* developed a PABA-based potentiometric sensor for detection of saccharides using the potential shift of PABA as the transduction principle [86, 87]. Therefore, we expected a decrease of the oxidation current and a concomitant positive shift in the peak potential upon dopamine binding. However, our data does not show appreciable shifts in E_pa_ upon dopamine binding. This is likely due to the low concentration of dopamine (nanomolar range) used in this study: the formation of a small amount of anionic ester could not induce an observable potential shift. In fact, both a decrease in the faradic current of the anodic peak as well as an increase in the potential of the anodic peak was observed when 1 μM dopamine was added to the electrochemical cell.

The faradic current of the anodic peak (I_PBS_) at E=0.45 V was recorded from the last cycle of CV curves at which the composite is stabilized in PBS solution, and the faradic current in the last CV cycles of each dopamine addition (I_DA_). Figure 5b shows the relative decrease of the faradic current of the composite in PBS as a function of the concentration of dopamine. The equation that was used to calculate each of the data points is shown as an inset in this figure. The correlation curve demonstrates an initial increasing linear region followed by a relatively horizontal regime. The linear portion, which extends from 1 nM to 10 nM, is very reproducible and has a very small standard deviation (standard error bars displayed on the curve; n=5). The theoretical detection limit (defined as the concentration that generates a signal three times larger than the noise) of this composite towards dopamine using cyclic voltammetry is 0.6 nM. Above the concentration of 30 nM the horizontal regime predominates: error bars for data points in this region are large because the saturation limits were quite different for different films. We do not know the exact reason yet at present, but we think that the saturation limits depend on the degree of aggregation of the ss-DNA-wrapped carbon nanotubes during deposition and drying on the electrode surfaces, which consequently influenced the amount and the quality of the PABA deposited on the electrode. Since the linear sensing range is in the appropriate regime for studying *in vivo* dopamine levels, our method holds great potential for molecular diagnosis of Parkinson's disease.

**Figure 6.**
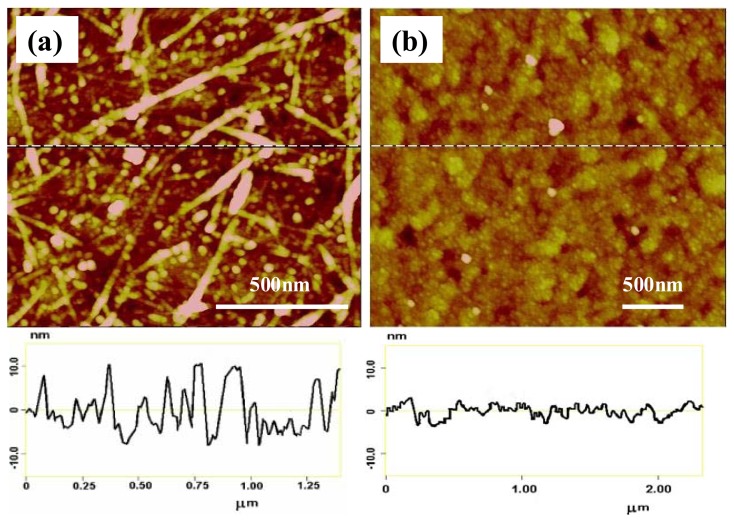
TappingMode AFM surface scans of electrochemically generated: (a) PABA/ss-DNA/SWNT composite film and (b) PABA film on gold electrodes.

The sensitivity towards dopamine increased by 10^4^ compared to the previous report in which neat PABA was used to modify the electrodes in a detection platform of micro-electrochemical transistors [43]. We believe that the ss-DNA/SWNTs in the composite increased the effective electrode surface area, causing a higher density of boronic acid groups available for dopamine binding and thus significantly enhanced the detection sensitivity. TappingMode AFM images (Figures 6a, 6b) clearly illustrate that the PABA/ss-DNA/SWNT composite film has a much greater surface roughness than the neat PABA film. The enhanced detection sensitivity might also be due to the improved electrochemical activity of the PABA in the composite, as described above.

We should mention that even higher detection sensitivity can be reached by optimizing the detection techniques. For example, a micro-electrochemical transistor as a detection platform normally has higher sensitivity because the conductivity of polyaniline can be changed by many orders of magnitude when its redox states are switched [51, 60, 88-90]. This large change in conductance of the polymer leads to amplification of the detection signal. Another technique, differential pulse voltammetry (DPV) [91], can greatly decrease the background charging currents, and in turn also increase the detection sensitivity. Our preliminary study demonstrates that the detection limit can be improved by two orders of magnitude when DPV instead of CV was applied to detect dopamine.

**Figure 7.**
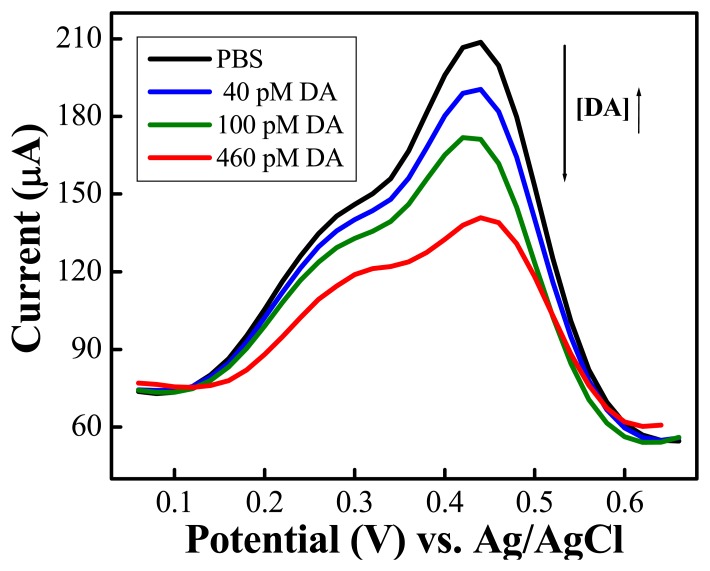
Differential pulse voltammograms of the composite in PBS and upon addition of different concentrations of dopamine. Potential scan rate: 100 mV·s^-1^; pulse amplitude: 50 mV; pulse width: 50 s. (Reproduced with permission from the American Chemical Society [48].)

Figure 7 shows that 40 pM dopamine (the smallest concentration tested) induces a considerable decrease in the anodic current in DPV curves, and that the current decrease is proportional to the concentration of dopamine introduced into the electrochemical cell. Moreover, the theoretical detection limit for dopamine detection using the DPV technique is 16 pM, a considerable improvement over recent dopamine sensors.

### 2.3. Interference by Ascorbic Acid

As described earlier, ascorbic acid is the most severe interferent in the determination of dopamine due to the nature of the oxidative reaction of dopamine and ascorbic acid on the electrode. In the non-oxidative detection scheme described herein, dopamine was not directly oxidized on the electrode. Therefore, the interference by AA should be inherently avoided [92, 93]. Indeed, it was reported that the AA interference was largely eliminated at a PABA-electrode dopamine sensor due to the high binding affinity between dopamine and the boronic acid moieties of PABA [43].

**Figure 8.**
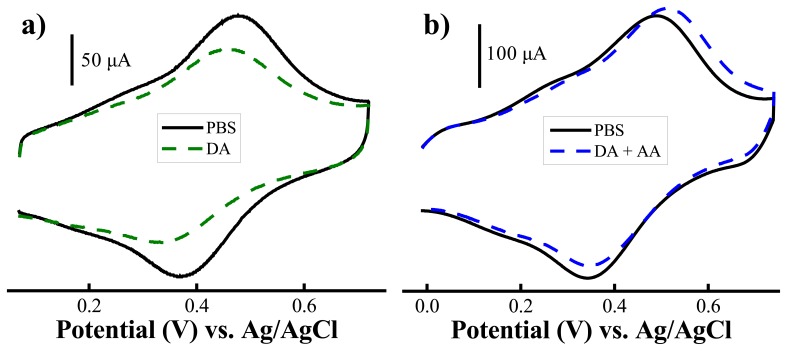
Cyclic voltammetric curves of the ss-DNA/SWNT/PABA modified Au electrodes in pH 7.4 PBS and upon addition of (a) 10 nM dopamine and (b) 10 nM dopamine and 0.15 mM ascorbic acid. Potential scan rate: 100 mV·s^-1^. (Reproduced with permission from the American Chemical Society [94].)

**Figure 9.**
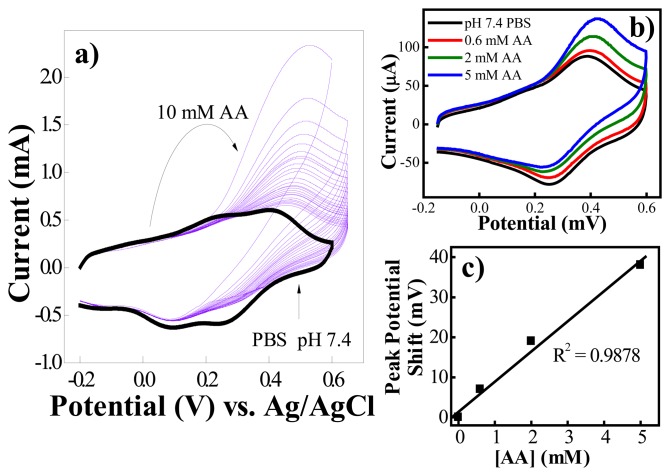
Influence of AA on the PABA/ss-DNA/SWNT composite sensor: a) CVs of the composite in PBS before (—) and after (—) addition of 10 mM ascorbic acid (the first CV upon addition of AA is the tallest one, and each successive cycle yielded a smaller curve); b) titration of the composite with AA ranging from 0.6 mM to 5 mM; c) a graph of the shift in peak potential of the composite's CV upon addition of AA as a function of the concentration of AA. Potential scan rate: 100 mV·s^-1^. (Reproduced with permission from the American Chemical Society [94].)

Surprisingly, we found that AA still severely interferes in the detection of dopamine, but with a different interference mechanism. Figure 8 shows that addition of 0.15 mM AA to 10 nM dopamine resulted in an increase of the oxidation current and positive shift of the oxidation potential (0.15 mM AA was used in this study because it is close to the physiological levels present in the extracellular space of the brain [95].) To understand the interference mechanism of AA we studied the electrochemical behavior of the PABA/ss-DNA/SWNT composite upon introducing AA alone to the electrochemical cell, and the results are displayed in Figure 9a. It is clear that introducing AA to the electrochemical cell triggered an extremely large initial oxidation current and a decrease of the corresponding reduction current of the polyaniline backbone. This is a typical electrochemical response characteristic of electrocatalytic reduction behavior of AA towards the polyaniline backbone [96]. Briefly, polyaniline was oxidized to its fully oxidized form, pernigraniline, when sweeping the potential in the positive direction in the CV experiment. Due to the strong reductive ability of the AA, the fully oxidized pernigraniline was reduced to the fully reduced state of the polymer backbone, leucoemeraldine, which became available again for oxidation in a larger quantity, thereby giving rise to the large electrocatalytic oxidation current during the subsequent sweep in the positive potential direction. In our experiment, further cycling caused the oxidation current to decrease rapidly and then the current stabilized at a value slightly higher than before addition of AA (Figures 8b and 9a). Note that the electrocatalytic reduction of the polyaniline backbone with ascorbic acid did not usually cause an oxidation potential shift and a decrease in the oxidation current with cycles [96]. Both phenomena were observed in this process, indicating that another chemical process occurred along with the electrocatalytic process.

Freund and co-workers reported that the oxidation potential of the PANI backbone shifts positively when a diol binds to the boronic acid groups along the backbone of PABA. Therefore the positive shift of the oxidation potential and the decrease of the oxidation current may be understood as a result of the formation of boronate ester complexes between AA and the boronic acid groups in the PABA/SWNT composite. By adding different concentrations of AA into the electrochemical cell, we found that both the oxidation current and the potential increased after the CV curves were stabilized (Figure 9b), and the positive potential shift increased monotonically as a function of the AA (Figure 9c). These results are consistent with the reports by Freund *et al.*, suggesting that binding occurs between ascorbic acid and the boronic acids on the PABA. The formation of the anionic ester between AA and the boronic acid groups in the PABA/SWNT composite reduced the K_a_ of the protonated quinone diimine groups in the polyaniline backbone, and reduction of the K_a_ caused a positive shift in the potential of the electrochemical conversion of emeraldine to penigraniline. To further confirm this conclusion and to further study the binding affinities of dopamine and ascorbic acid with the boronic acid groups along the polyaniline backbone, a fluorescence binding assay was utilized to measure the association constants.

### 2.4. Further study of the interference of ascorbic acid: a fluorescence binding assay

**Scheme 3.**
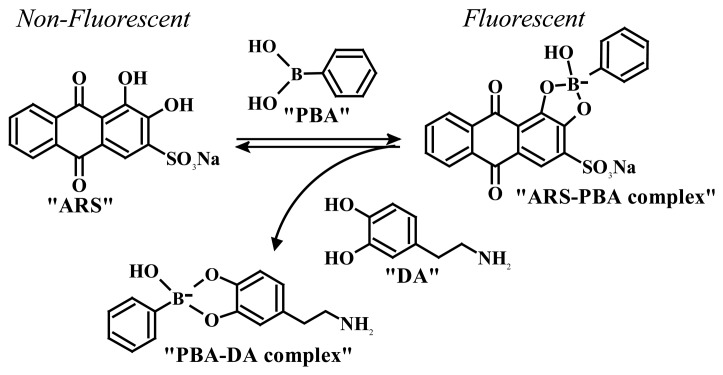
Fluorescence protocol for determining the binding constant between boronic acids and diols using the reporter dye ARS. (Reproduced with permission from the American Chemical Society [94].)

Springsteen *et al.* developed a general method for measuring association constants of diol-boronic acid complexes under physiological pH solutions [97, 98]. This protocol is a three-component competitive assay containing a fluorescent reporter dye, Alizarin Red S (ARS), phenylboronic acid (PBA), and the diol-containing compound of interest. ARS has a diol group and is able to form a boronate ester with PBA. The free ARS is only weakly fluorescent because the excited state proton transfer from the phenol hydroxyl group of ARS to the ketone oxygen results in the fluorescence quenching. The fluorescence of ARS increases upon formation of the boronate ester with PBA because the fluorescence quenching mechanism is removed. The binding constant between the diol of interest and PBA is determined based on the competitive binding of the ARS and the diol to PBA (Scheme 3).

When the diol binds to the PBA it disrupts the ARS-PBA complex, thereby decreasing the concentration of the ARS-PBA complex as well as the fluorescence signal of the solution. This protocol requires knowledge of the binding constant between the PBA and the ARS, which can be determined using the Benesi-Hildebrand method [99]. The binding constant between PBA and the diol (DA and AA in this work) is calculated by determining the concentration of PBA displaced from the PBA-ARS complex upon addition of various concentrations of the diol [98]. Note that we could not directly measure the binding constants of DA or AA to the boronic acid groups in the PABA composite using this method because of the possible quenching ability of the carbon nanotubes in the composite and the relative difficulty in determining the concentration of the boronic acid moieties in the composite. This concentration is required in the calculations for the protocol. Considering that the repeating unit of the PABA in the composite is essentially phenylboronic acid, the measured binding constant between PBA and DA (or AA) could indicate the relative binding strength of the DA (or AA) to the boronic acid groups in the PABA composite, although the absolute values may be slightly different.

Solutions of 9 μM ARS, 9 μM ARS and 2 mM PBA, and 9 μM ARS and 2 mM PBA with a range of DA or AA concentrations were prepared in 0.10 M phosphate buffer (pH 7.4). They were allowed to react for 5 minutes at room temperature before performance of the fluorescence experiments. The solutions were excited at 468 nm and the fluorescence intensities were monitored at the emission wavelength of 588-590 nm. Equations use to calculate the binding constants are shown below [100]:

(1) $$Q=\frac{\left[\text{ARS}\right]}{\left[\text{ARS}-\text{PBA}\right]}$$

(2) $$\frac{\left[\text{diol}\right]}{P}=\frac{{\text{K}}_{\text{eq}}}{{\text{K}}_{\text{a}}}Q+1$$

(3) $$P=[{\text{diol}}_{\text{o}}]-\frac{1}{Q{\text{K}}_{\text{eq}}}-\frac{\left[{\text{ARS}}_{\text{o}}\right]}{Q+1}$$

where K_eq_ is the association constant of the ARS-PBA complex (determined by the Benesi-Hildebrand method), K_a_ is the association constant of the boronic acid–diol complex, [diol_o_] is the total diol concentration, [ARS_o_] is the total ARS concentration, *Q* is the ratio of the concentration of uncomplexed ARS to complexed ARS (Equation 1), and *P* is defined by Equation 3. The K_a_ of the boronic acid–diol complex was determined by plotting [diol]/P vs. Q, and dividing K_a_ by the slope of the plot, as per Equation 2. The fluorescence emission spectra were obtained at a Cary Eclipse fluorescence spectrophotometer (Varian).

**Figure 10.**
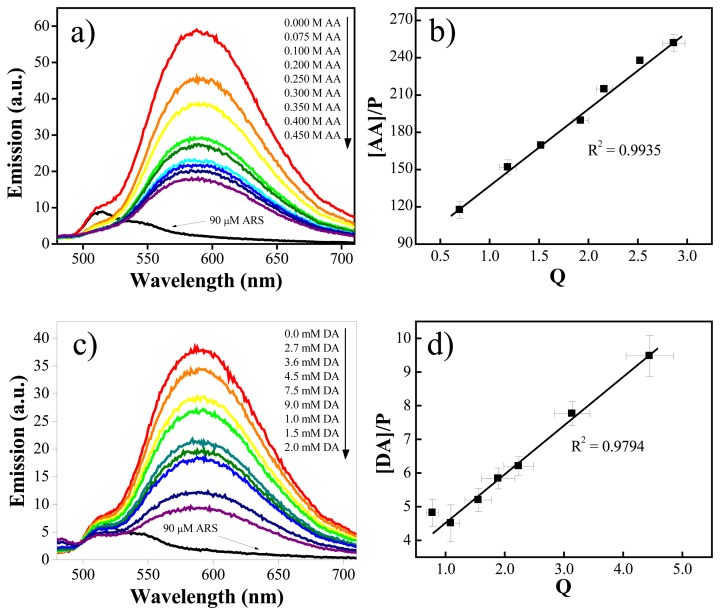
Fluorescent binding assay results of the affinity between PBA and AA and between PBA and DA: a) Fluorescence emission curves of the PBA-ARS complex upon titration with a range of AA concentrations; b) Linear correlation between [AA]/P and Q; c) Fluorescence emission curves of the PBA-ARS complex upon titration with a range of DA concentrations; d) Linear correlation between [DA]/P and Q. (Reproduced with permission from the American Chemical Society [94].)

Figure 10c shows the fluorescence of the ARS-PBA complex upon addition of different concentrations of dopamine. The fluorescence decreases as a function of the dopamine concentration, as expected. We calculated the binding constant between PBA and dopamine as 890 ± 42 M^-1^ (mean ± SEM). Wang *et al.* determined the binding constant between PBA and catechol to be 830 M^-1^. As catechol is very structurally similar to dopamine (although it does not contain an ethylamine group like dopamine), the small difference in their K_a_ values is understandable. However, it is necessary to mention the possibility that the existence of free amine group in dopamine may quench the fluorescence of the ARS-PBA complex due to the lone electron pair on the amine nitrogen [101]. This would result in an apparently larger calculated binding constant compared to catechol's, which only contains a diol group [98].

To elucidate the influence of the amine group in dopamine on the fluorescence signal during the binding constant measurement, a control experiment was performed with tyramine. Tyramine is also a neurotransmitter and it has a very similar molecular structure as dopamine, but without a diol group to bind boronic acid: rather, it possesses a single alcohol group and an ethylamine group in the *para* position. We utilized the same fluorescence binding assay to study how the amine group interacts with the PBA-ARS complex by monitoring the fluorescence signal upon addition of different concentrations of tyramine into the PBA-ARS complex solution. We found that addition of tyramine barely changed the fluorescence signal of the PBA-ARS complex, suggesting that possible quenching of the ARS-PBA complex by the free amine groups of dopamine, resulting in an overestimate of the calculated PBA-DA binding constant, is negligible.

**Scheme 4.**
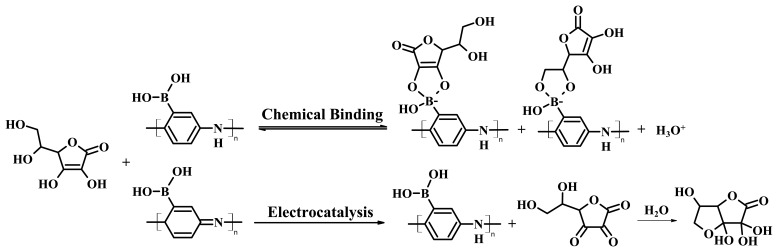
Molecular basis for the interactions between PABA and AA. (Reproduced with permission from the American Chemical Society [94].)

The aforementioned fluorescence-based binding assay was also employed to calculate the binding affinity of ascorbic acid to PBA, the value of whose binding constant is 21 ± 1.8 M^-1^ (mean ± SEM), which is approximately 40-fold lower than the DA association constant (890 ± 42 M^-1^). Considering that the concentration of AA is three or four orders of magnitude higher than the concentration of DA in physiological samples, large amounts of AA can therefore bind to the boronic acid groups along the polyaniline backbone under physiological conditions. Therefore, the interference by AA toward the detection of dopamine is a two-pronged problem in this non-oxidative approach. On one hand, the electrocatalytic reductive ability of AA caused a large increase of the oxidation current of the polyaniline backbone, and on the other hand AA chemically bonded to the boronic acid groups, which induced a decrease of the oxidation current and a positive shift of the oxidation potential. The net effects of these two divergent factors determine the degree of AA interference on the detection of DA. The chemical and electrochemical interactions between PABA and AA are summarized in Scheme 4, which may serve as a molecular paradigm for the interference of AA towards other PABA-, PANI-, and boronic acid-based sensors.

Although we still do not understand why the current approach is contradictory to the previous reports about AA interference, we speculate that one of the most important reasons is the extremely high sensitivity provided in the current sensing approach, which “detected” the previously undetectable AA, leading to the observed interference. Finally it is important to mention that a freshly prepared ascorbic acid solution is required to study the interference effect of AA. We noticed that the AA solutions that were used approximately one day after preparation did not demonstrate interference. We understand that this is because AA is not stable in solution [102]. *In vivo* AA is protected by chemical interactions with physiological proteins but in vitro AA is susceptible to oxidation, which is not surprising considering that the foremost chemical role of Vitamin C is as a reducing agent. It is reported that measurable oxidation of AA occurs within hours [102]. The oxidized product of AA is dehydroascorbic acid [103], which is not electrochemically active and its binding to boronic acid is extremely weak [104].

### 2.5. Elimination of the Ascorbic Acid Interference with Nafion

Several methods have been reported to eliminate the interference of AA towards the determination of dopamine, such as use of the enzyme ascorbate oxidase (AOx). Selective oxidation of AA by AOx has been widely used in the development of selective biosensors, including those for glucose, lactose, and dopamine [105-107]. The oxidized AA after hydrolysis is not electrochemically active, thereby eliminating the interference of AA towards electrochemical biosensors. Other approaches include using charge-discriminating membranes to preferentially accumulate the positively charged dopamine (pK_a_ 8.9) and reject the negatively charged ascorbate (pK_a_ 4.2) at the electrode surfaces in physiological pH. The widely used perfluorosulfonated polymer Nafion, a cation-exchange polymer, repels ascorbate and other anions and can provide a transport channel solely for cations. Due to their biocompatibility, Nafion films have been extensively employed for the modification of electrode surfaces and for the construction of amperometric biosensors [108, 109]. Towards the aim of *in vivo* detection of DA, we deposited a thin layer of Nafion on top of the PABA/SWNT composite to diminish the ascorbate interference.

**Figure 11.**
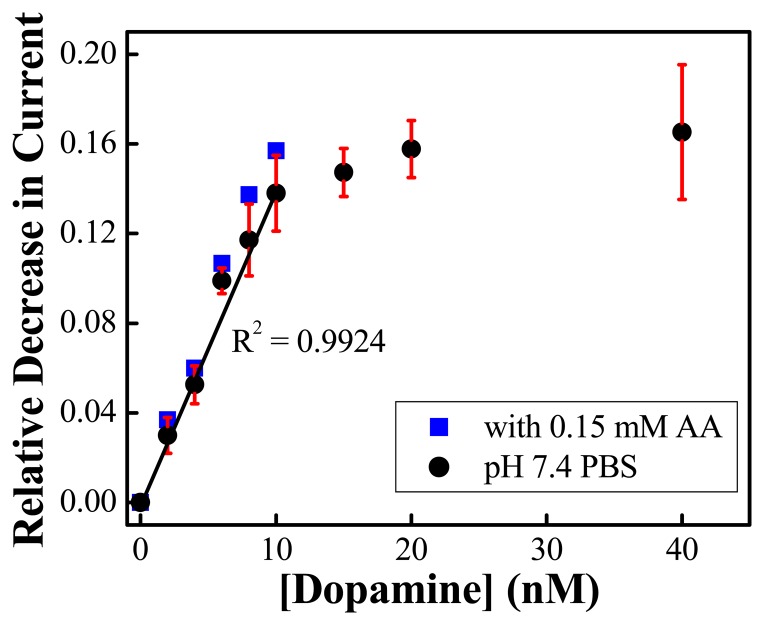
Correlation curves for the detection of dopamine on the electrode modified with ss-DNA/SWNT/PABA/Nafion composite in the absence (●) and presence ( 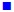) of 0.15 mM AA (*n*=3) (Reproduced with permission from the American Chemical Society [48].)

Deposition of a layer of Nafion does not alter the redox activity of the ss-DNA/SWNT/PABA film in neutral pH solutions. The CV curves are very similar to the curves shown in Figure 5. There is no indication of electrocatalytic reduction of AA occurring on the electrode, indicating that the Nafion film is able to effectively block the ascorbate from interacting with the ss-DNA/SWNT/PABA film. Figure 11 shows the calibration curves of dopamine in the absence and presence of 0.15 mM acscorbic acid. In absence of ascorbic acid, it can be seen that that dopamine calibration curve follows the same shape as the binding curve described in Figure 5b, which was obtained on the electrodes without the Nafion layer.

The correlation coefficient for the reproducible detection of dopamine is 0.9924, and the detection limit (1.5 nM) is slightly higher than that without Nafion (0.6 nM). This is perhaps due to Nafion behaving as a diffusive barrier, i.e. the Nafion film decreased the amount and/or the speed of dopamine diffusing to the ss-DNA/SWNT/PABA film. With 0.15 mM ascorbic acid in the dopamine solutions, the data points in the calibration curve of dopamine reside in the positive error region of the calibration curve for dopamine without ascorbate present. These results demonstrate the near-complete elimination of interference by ascorbic acid. The strong cation exchange of Nafion leads to an uptake of the positively changed dopamine and the anionic ascorbate interferent is electrostatically rejected from the surface because of the negatively charged Nafion. Since the linear correlation is between 1 to 10 nM dopamine, which is an appropriate concentration range for Parkinson's disease patients [2, 13, 14], this approach holds great potential for molecular diagnosis of Parkinson's disease.

## 3. Conclusions

In this review we have summarized our efforts in electrochemical detection of dopamine with high sensitivity and selectivity by modifying the electrode surface with a thin layer of *in-situ* polymerized poly(anilineboronic acid)/carbon nanotube composite and a thin layer of the highly permselective Nafion film. Since direct oxidation of dopamine is avoided in this approach, the associated problems with direct oxidation, such as electrode fouling and dopamine regeneration, were prevented. Furthermore, the DNA-wrapped single-walled carbon nanotubes in the composite not only greatly improved the electrochemical activity of the composite in physiologically relevant solutions, they also increased the effective electrode surface area, and therefore the density of boronic acid groups available for binding dopamine. These features significantly enhanced the sensitivity for dopamine detection. Dopamine concentrations as low as 1 nM were detected with cyclic voltammetry, and the electrochemical current change was linear in the range of 1–10 nM. Optimization of the detection technique resulted in a detection limit of 16 pM, which is six magnitudes lower than that of the sensor in which PABA alone was used for the detection. In this work, we also found that ascorbic acid interfered with the detection if Nafion was not deposited on the electrode, which is contrary to previous reports of nonoxidative PABA-based sensors. We studied the mechanism of interference by ascorbic acid and the results show that the interference mechanism is very different from the approaches relying on direct oxidation of dopamine at the electrode. The ascorbic acid was able to electrocatalytically reduce the fully oxidized polyaniline backbone during the electrochemical oxidation process. The ascorbic acid was also able to bind to the boronic acid groups through its planar diol as dopamine does, although its binding affinity is lower. Coating a thin layer of Nafion on top of the composite eliminated the interference from ascorbic acid. The strong cation exchange of Nafion leads to an uptake of positively charged DA and the negative charges on the Nafion film electrostatically rejected the ingress of the negatively charged ascorbate to the PABA/carbon nanotube composite. The sensitivity of the sensor along with its improved selectivity might allow for its potential use in the diagnosis of Parkinson's disease. A clear understanding of the dopamine transduction mechanism and AA interference mechanism in this non-oxidative approach is essential to eliminate other interferences toward *in vivo* and *in vitro* detection of dopamine, which is the long term goal of our continuing efforts.
